# Transcriptome-Based Identification of Differently Expressed Genes from *Xanthomonas oryzae* pv. *oryzae* Strains Exhibiting Different Virulence in Rice Varieties

**DOI:** 10.3390/ijms17020259

**Published:** 2016-02-19

**Authors:** Tae-Hwan Noh, Eun-Sung Song, Hong-Il Kim, Mi-Hyung Kang, Young-Jin Park

**Affiliations:** 1 National Institute of Crop Science, Rural Development Administration, Wanju-gun 55365, Korea; nohtw831@korea.kr (T.-H.N.); kangmh@korea.kr (M.-H.K.); 2 National Institute of Agricultural Science, Rural Development Administration, Jeonju 54874, Korea; ses5494@korea.kr; 3 Department of Biomedical Chemistry, Konkuk University, Chungju 27478, Korea; kwangdae7@kku.ac.kr

**Keywords:** bacterial blight, differently expressed gene, transcriptome, virulence, *Xanthomonas oryzae* pv. *oryzae*

## Abstract

*Xanthomonas oryzae* pv. *oryzae* (*Xoo*) causes bacterial blight (BB) in rice (*Oryza sativa* L.). In this study, we investigated the genome-wide transcription patterns of two *Xoo* strains (KACC10331 and HB1009), which showed different virulence patterns against eight rice cultivars, including IRBB21 (carrying *Xa21*). In total, 743 genes showed a significant change (*p-*value < 0.001 in *t*-tests) in their mRNA expression levels in the HB1009 (K3a race) strain compared with the *Xoo* KACC10331 strain (K1 race). Among them, four remarkably enriched GO terms, DNA binding, transposition, cellular nitrogen compound metabolic process, and cellular macromolecule metabolic process, were identified in the upregulated genes. In addition, the expression of 44 genes was considerably higher (log2 fold changes > 2) in the HB1009 (K3a race) strain than in the *Xoo* KACC10331 (K1 race) strain. Furthermore, 13 and 12 genes involved in hypersensitive response and pathogenicity (*hrp*) and two-component regulatory systems (TCSs), respectively, were upregulated in the HB1009 (K3a race) strain compared with the *Xoo* KACC10331 (K1 race) strain, which we determined using either quantitative real-time PCR analysis or next-generation RNA sequencing. These results will be helpful to improve our understanding of *Xoo* and to gain a better insight into the *Xoo*–rice interactions.

## 1. Introduction

Bacterial blight (BB) has become one of the most devastating diseases of rice and an important problem affecting rice production worldwide. Some bactericides have been developed to control the disease, but none is highly effective. Thus, genetic basis breeding for BB resistance has been found to be the most effective method for the control of BB [1]. At least 38 BB resistance (*R*) genes in rice have been identified [2,3], of which six (*Xa2*, *Xa4*, *Xa7*, *Xa30*, *Xa33*, and *Xa38*) have been physically mapped and eight (*Xa1*, *xa5*, *xa13*, *Xa21*, *Xa26/Xa3*, *Xa10*, *Xa23* and *Xa27*) have been cloned [2,3,4,5,6,7,8,9,10,11]. Among them, the *Xa21* gene was identified in the wild species *Oryza* *longistaminata* and shown to be highly effective against BB races of South and Southeast Asia [12]. Pattern recognition receptors (PRRs) are proteins of critical importance in detecting invading pathogens in plants and animals. In rice, *XA21* is a well-characterized PRR and is a leucine-rich repeat receptor-like kinase that confers broad-spectrum resistance to multiple strains of the bacterial pathogen *Xoo*. Several bacterial genes that are required for activation of *XA21*-mediated immunity (*rax* genes) have been identified [13,14,15]. Studies by Lee *et al.* [16] on XA21-mediated immunity activation showed that a mutation in the *Xoo* protein (PXO_03968), a 198-aa-long protein named Ax21 (for Activator of XA21-mediated immunity), allowed the bacterium to evade XA21-mediated immunity. However, further analysis of this gene revealed that a mutation in *ax21* (PXO_03968) still activated XA21-mediated immunity [17].

Korean *Xoo* isolates have been classified into five races (K1 to K5) based on the *Xoo* differential system with five rice cultivars [18]. Recent pathotype analysis of the Korean *Xoo* isolates revealed that the K1 race has decreased, whereas the K2 and K3 races have increased in Korea [19,20,21]. In addition, Noh *et al.* [22] reported that a new *Xoo* race (K3a) is pathogenic for rice cultivars, possesses the *Xa1,* *Xa3,* or *Xa4* gene according to a virulence assay, and caused serious damage to rice production in the southwestern coastal areas of Korea in 2003 [22]. Recently, Song *et al.* [23] reported that the AFLP-derived marker could be used for the specific detection of the K3a race. This marker is important because early identification of this race by rapid methods is important for assessing the health status of rice. However, fundamental studies on the properties of this new race remain challenging.

These two aspects, *i.e.*, that Ax21 is not the activator of XA21-mediated immunity and the reports regarding the new *Xoo* race (K3a) outbreak, motivated us to carry out more detailed studies based on genome-wide transcription analysis with *Xoo* KACC10331 (virulent against IRBB21) and *Xoo* HB1009 (K3a race). Here, we report the genome-wide transcription profiling of genes in two *Xoo* strains, one (K1 race) is virulent against IRBB21 (carrying *Xa21*) and the other is a new *Xoo* race (K3a) that is pathogenic for rice cultivars and possesses the *Xa1*, *Xa3*, or *Xa4* gene.

## 2. Results

### 2.1. Virulence of the Xoo K1 Race and K3a Race Strains

**Figure 1:**
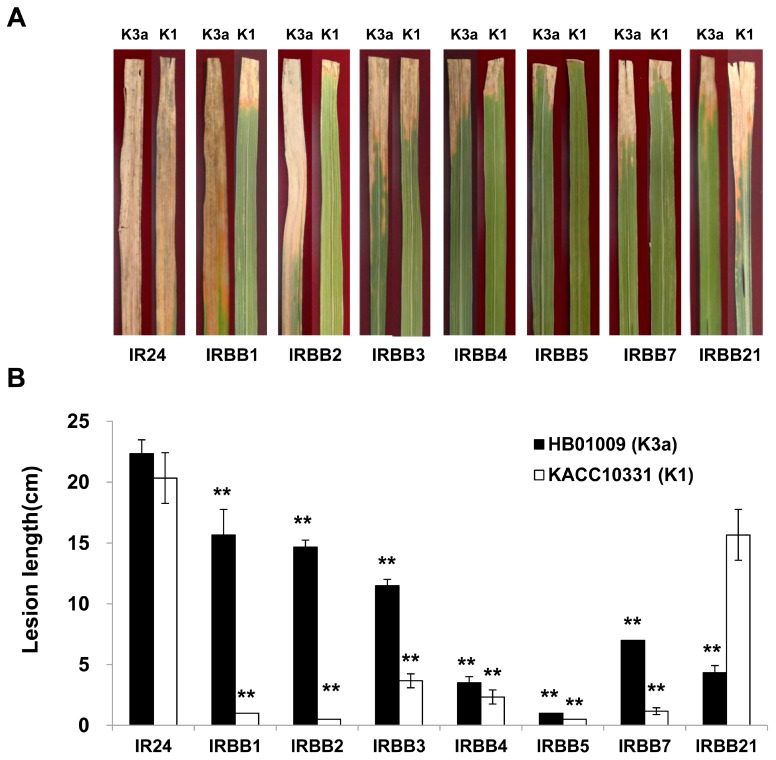
Virulence assay (**A**) and lesion length (**B**) of rice varieties inoculated with two *Xanthomonas oryzae* pv. *oryzae* strains (K1 and K3a race). Asterisks represent statistically significant differences relative to a susceptible rice cultivar (IR24) (paired, two-tailed Student’s *t* test, ** *p*-value < 0.01).

In a virulence assay, one susceptible rice cultivar (IR24) and seven near-isogenic lines (NILs) against bacterial blight, including IRBB1 (carrying *Xa-1*), IRBB2 (carrying *Xa-2*), IRBB3 (carrying *Xa-3*), IRBB4 (carrying *Xa-4*), IRBB5 (carrying *Xa-5*), IRBB7 (carrying *Xa-7*), and IRBB21 (carrying *Xa-21*), from the International Rice Research Institute (IRRI), were inoculated with the *Xoo* KACC10331 (K1 race) and HB1009 (K3a race) strains. There were no significant differences between the lesion lengths of three varieties (IR24, IRBB4, and IRBB5) when inoculated with the two strains. However, the two strains showed different virulence patterns against the rice varieties IRBB1, IRBB2, IRBB3, IRBB7, and IRBB21. Twenty-one days after inoculation, the average lengths of the lesions caused by the KACC10331 (K1 race) strain were 1 ± 0, 0.5 ± 0, 3.7 ± 0.6, and 1.2 ± 0.3 cm against IRBB1, IRBB2, IRBB3, and IRBB7, respectively, whereas those of the lesions caused by the HB1009 (K3a race) strain were 15.6 ± 2, 14.6 ± 0.5, 11.5 ± 0.5, and 7.0 ± 0 cm, respectively (Figure 1). In contrast, for IRBB21, the average lesion length caused by the KACC10331 (K1 race) strain was 15.7 ± 2.0 cm, whereas that of the lesion caused by the HB1009 (K3a race) strain was 4.3 ± 0.5 cm (Figure 1).

Our results indicated that the virulence of the KACC10331 (K1 race) strain was significantly weaker than that of the HB1009 (K3a race) strain when infecting four rice varieties (IRBB1, IRBB2, IRBB3, and IRBB7). However, the virulence of the KACC10331 (K1 race) strain was significantly stronger than that of the HB1009 (K3a race) strain when infecting IRBB21 (carrying the *Xa-21* resistance gene) NIL.

### 2.2. RNA-seq Reads Mapping and GO Term Enrichment of Differentially Expressed Genes

**Figure 2:**
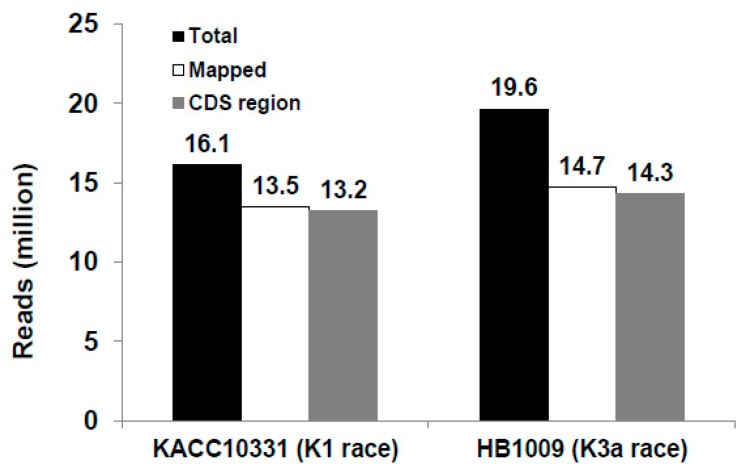
Total number of R-seq reads from each *Xanthomonas* *oryzae* pv. *oryzae* strain library and mapped reads to the *Xanthomonas* *oryzae* pv. *oryzae* KACC10331 genome.

To compare the transcriptome profiles of the *Xoo* KACC10331 (K1 race) and HB1009 (K3a race) strains, RNA sequencing libraries were constructed. The two libraries generated about 16.1 and 19.6 million reads, which were mapped to the *X. oryzae* pv. *oryzae* KACC10331 genome sequence in the NCBI GenBank database (NC_006834.1) with 96.9% and 96.4% matched reads to NCBI annotated gene regions (Figure 2). There were 4065 protein-coding genes (CDS) among 4281 annotated genes in the genome of KACC10331 by genome analysis [24]. A total of 4062 and 3995 protein-coding genes were detected (≥5 mapped reads) in the *Xoo* KACC10331 (K1 race) and HB1009 (K3a race) strains, respectively (Figure 2).

**Figure 3:**
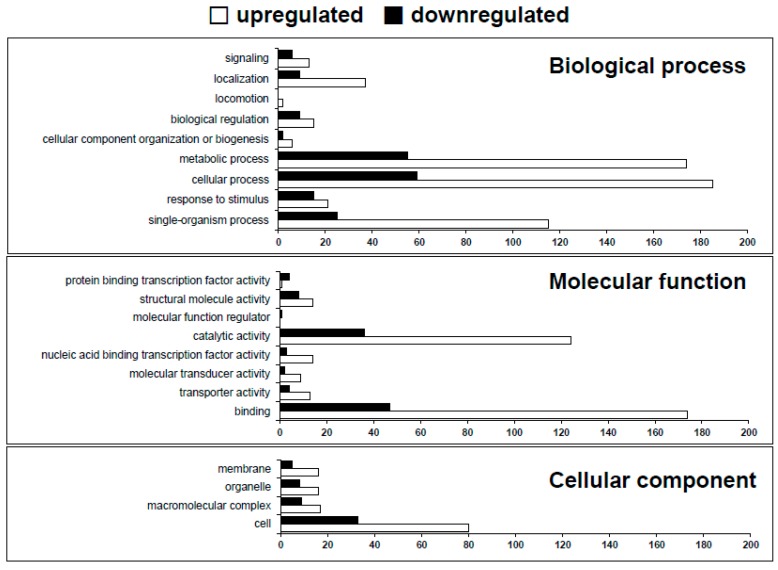
Functional categorization of the upregulated and downregulated genes in *Xanthomonas oryzae* pv. *oryzae* HB1009 (K3a race) based on the GO annotation.

To identify DEGs, we used the R-package DEGseq [25]. Genes with significant differences in expression level between the two strains were evaluated using the criterion of a *p*-value less than 0.001 in a *t*-test, resulting in 743 DEGs when the HB1009 (K3a race) strain was compared with the *Xoo* KACC10331 strain (K1 race) (Table S1). To further analyze these DEGs, the transcripts were categorized according to their annotated function with respect to biological processes, molecular functions, and cellular components, on the basis of the blast and GO term annotation using Blast2GO software [26]. The biological processes mediated by these DEGs were primarily associated with cellular processes, metabolic processes, and single organism processes, among others (Figure 3, Table S2). The most major molecular functions were binding and catalytic activity. Cell was the most majority in the cellular component category. Most of these categories contained a larger numbers of genes with typically upregulated expression than those of genes with typically downregulated expression (Figure 3, Table S2). In the GO term enrichment analysis using Blast2GO (Fisher’s exact test), four remarkably enriched GO terms, DNA binding, transposition, cellular nitrogen compound metabolic process, and cellular macromolecule metabolic process, were identified in the upregulated genes. However, there were no GO terms enriched in the genes with downregulated expression (Table S3). Thus, the RNA-seq and GO term enrichment analysis results suggested that some of these genes were predominantly expressed in HB1009 (K3a race), since the expression of 44 genes (a total of 147 enriched genes) was considerably higher (log2 fold changes > 2) in the HB1009 (K3a race) strain than in the *Xoo* KACC10331 (K1 race) strain (Table S4).

### 2.3. Differential Expression of hrp Genes between the Xoo K1 Race and K3a Race Strains

**Figure 4:**
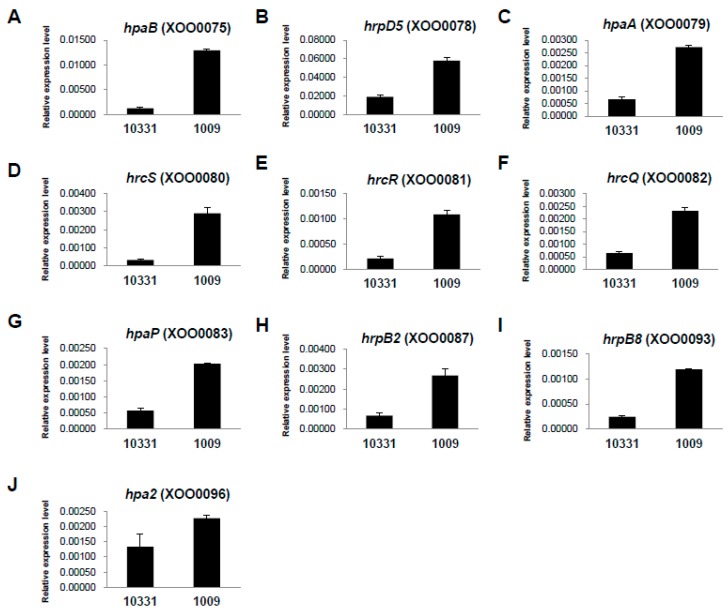
The expression patterns of *hrp* genes of the *Xanthomonas* *oryzae* pv. *oryzae* KACC10331 (K1 race) and HB1009 (K3a race) strains. The gene expression levels (arbitrary units) of *hpaB* (**A**); *hrpD5* (**B**); *hpaA* (**C**); *hrcS* (**D**); *hrcR* (**E**); *hrcQ* (**F**); *hpaP* (**G**); *hrpB2* (**H**); *hrpB8* (**I**); and *hpa2* (**J**) were normalized using 16S RNA as an internal reference. Gene expression levels were quantified by real-time RT-PCR.

In xanthomonads, the *hrp* (hypersensitive response and pathogenicity) genes are highly conserved and clustered, which is essential for the pathogenicity of *Xoo* [27,28]. According to the genome sequence of *Xoo* KACC10331, the *hrp* gene cluster was composed of nine *hrp* (*hrpF*, *hrpE*, *hrpD6*, *hrpD5*, *hrpB1*, *hrpB2*, *hrpB4*, *hrpB5*, and *hrpB7*), nine *hrc* (*hrp* conserved; *hrcS*, *hrcR*, *hrcQ*, *hrcV*, *hrcU*, *hrcJ* (*hrpB3*), *hrcN*, *hrcT* (*hrpB8*), and *hrcC*), and eight *hpa* (*hrp*-associated; *hpaF*, *hpaB*, *hpaA*, *hpaP*, *hpa1*, *hpa2*, *hpa3*, and *hpa4*) genes [24,27]. The expression of the *hrpA* and *hrpX* genes is thought to be controlled by HrpG, which is the OmpR family regulator [29]. In addition, *hrpX*, which belongs to the AraC family of positive transcriptional activators, controls the expression of operons *hrpB* to *hrpF* and a number of virulence factors [30]. In transcriptome analysis, 13 *hrp* (*hpa4*; XOO0074, *hpaB*; XOO0075, *hrpD5*; XOO0078, *hpaA*; XOO0079, *hrcS*; XOO0080, *hrcR*; XOO0081, *hrcQ*; XOO0082, *hpaP*; XOO0083, *hrpB2*; XOO0087, *hrpB8*; XOO0093, *hpaD6*; XOO0077, *hpa2*; XOO0096, and *hpa3*; XOO4700) were significantly upregulated in the HB1009 (K3a race) strain compared with the *Xoo* KACC10331 (K1 race) strain (Table 1, Figure 4). The log2 FCs of the 13 genes ranged from 0.97 to 2.47. Among them, the expression levels of four *hrp* genes, including *hpaB* (XOO0075), *hpaA* (XOO0079), *hrcS* (XOO0080), and *hrpB2* (XOO0087), were considerably higher (log2 fold changes > 2) in the HB1009 (K3a race) strain than in the *Xoo* KACC10331 (K1 race) strain (Table 1, Figure 4). Most of these upregulated genes involved in the *hrp* cluster are known to be crucial for the HR and virulence in *Xoo* [27]. In addition, Zhang *et al.* [31] reported that an increase in transcript alterations of *hrp* genes and type III effectors results in increased *Xoo* virulence. These results suggest that the increased virulence of the HB1009 strain compared with the KACC10331 strain when infecting some rice varieties was due to the differentially upregulated expression of *hrp* genes.

### 2.4. Differential Expression of Two-Component Systems between the Xoo K1 Race and K3a Race Strains

**Figure 5:**
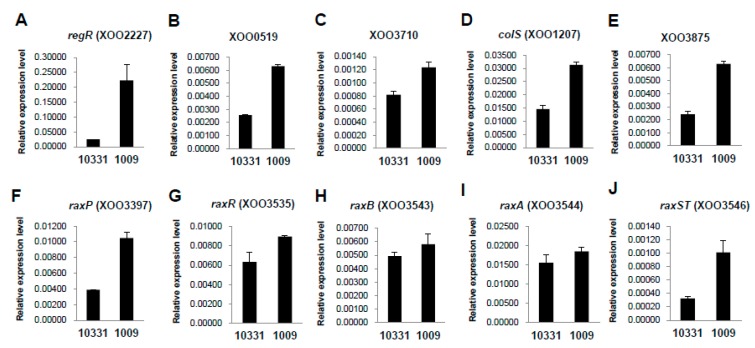
The expression patterns of TCS and *rax* genes of the *Xanthomonas oryzae* pv. *oryzae* KACC10331 (K1 race) and HB1009 (K3a race) strains. The gene expression levels (arbitrary units) of *regR* (**A**); XOO0519 (**B**); XOO3710 (**C**); *colS* (**D**); XOO3875 (**E**); *raxP* (**F**); *raxR* (**G**); *raxB* (**H**); *raxA* (**I**); and *raxST* (**J**) were normalized using 16S RNA as an internal reference. Gene expression levels were quantified by real-time RT-PCR.

To cope with various environmental conditions, including limited-nutrient niches and various toxic substances produced by the host, bacteria have evolved a mechanism involving two-component regulatory systems (TCSs). TCSs are composed of histidine kinases (HKs) and response regulators (RRs). In response to environmental stimuli, the HK phosphorylates the cognate RR, which then regulates gene expression [32]. In this study, among the 48 TCSs, 15 TCSs showed significantly different expression levels in the HB1009 (K3a race) strain compared with *Xoo* KACC10331 (K1 race). Twelve TCSs (XOO0336, XOO0519, XOO1105, XOO1207, XOO2227, XOO2228, XOO3659, XOO3709, XOO3710, XOO3871, XOO3875, and XOO4008) and three TCSs (XOO0423, XOO2322, and XOO2798) were upregulated and downregulated, respectively, in the HB1009 (K3a race) strain compared with the Xoo KACC10331 (K1 race) strain (Figure 5, Table 2, Table S1). In addition, three (XOO1105, XOO2227, and XOO4008) out of 12 significantly upregulated TCSs were over-represented in the GO term enrichment analysis (Table S4).

Quorum sensing (QS) is the process in which bacteria monitor their own population density by sensing the levels of extracellular signal molecules that are released by the microorganisms, and the QS systems might enable pathogens to overcome the host defense mechanisms [33]. RpfC and RpfG serve as a two-component system for the perception and transduction of the extracellular diffusible signal factor (DSF) family signal-mediated QS [34,35,36]. The expression pattern of the RpfC/RpfG two-component regulatory system (XOO2870 and XOO2871) associated with QS was different between the HB1009 and KACC10331 strains (Table S1). Furthermore, Ryan *et al.* [37] reported that several domain proteins, including GGDEF, EAL, and HD-GYP, are involved in a network of signal transduction systems for responding to different environmental factors to modulate the level of the second messenger cyclic di-GMP in *X*. *campestris* pv. *campestris*. In this study, a TCS regulatory protein with a GGDEF (XOO2787) or with both GGDEF and EAL domains (XOO0520) had upregulated (0.59 and 1.26 log2 FC, respectively) expression in the HB1009 strain. In contrast, a TCS regulatory protein with an HD-GYP domain (XOO2798) had significantly downregulated expression (−2.2 log2 FC) in the HB1009 strain compared with the KACC10331 strain. In addition, XOO2860 (encoding cyclic di-GMP phosphodiesterase A) had upregulated expression in the HB1009 strain compared with the KACC10331 strain (Table S2).

Rice lines carrying *Xa21* (encodes a leucine-rich repeat receptor-like kinase) are able to induce an effective defense response to multiple strains of the bacterial *Xoo* pathogens [38]. In addition, several genes that are required for activation of XA21-mediated immunity (*rax*) have been identified in *Xoo* [13,14,15]. Furthermore, Lee *et al.* [39] and Zhang *et al.* [31] reported that *Xoo* requires a regulatory TCS called RaxRH to regulate expression of 10 *rax* genes, including *raxA*, *raxB*, *raxC*, *raxST*, *raxP*, *raxQ*, *raxR*, *raxR2*, *raxH*, and *raxH2*. Our results revealed that all of these *rax* genes were not expressed at significantly different levels between the HB1009 and KACC10331 strains according to the DEGseq analysis (*p*-value > 0.001, FALSE), except for *raxR2* (XOO0423) (Table 2, Table S2). However, some of these genes, including *raxP* (XOO3397), *raxR* (XOO3535), *raxB* (XOO3543), *raxA* (XOO3544), and *raxST* (XOO3546), showed different expression patterns between the two strains in the qRT-PCR analysis (Figure 5). As described above, the expression of *raxR2* (XOO0423) associated with TCSs was significantly downregulated in the HB1009 (K3a race) strain (Table 2, Table S1), which showed reduced virulence against IRBB21 (carrying *Xa-21*) (Figure 1). In contrast, *raxR* (XOO3535) was upregulated in the HB1009 (K3a race) strain, although the *raxR* gene did not show significantly different expression levels in the DEGseq analysis (*p*-value > 0.001, FALSE) (Table 2, Table S2). The findings of the current study are consistent with those of Lee *et al.* [39] who reported that a response regulator encoded by *phoP* (*raxR2*; XOO0423) is upregulated in a *raxR* gene knockout mutant strain. The PhoPQ-regulated protein (XOO1731) not only is required for AvrXa21 activity but also regulates various cellular activities as a regulator of virulence in *Salmonella* and other species [40,41,42]. Upregulation of the phoPQ-regulated protein was detected in the HB1009 strain, although this gene did not show significantly different expression levels in the DEGseq analysis (*p*-value > 0.001, FALSE).

## 3. Discussion

In the past several years, a large number of *Xoo* pathogenesis-related genes have been isolated and characterized, as many researchers have tried to elucidate the mechanisms of the *Xoo*–rice interaction. However, many aspects of the mechanisms of the *Xoo*–rice interaction are still not clearly understood. For example, although several genes involved in the activation of *XA21*-mediated immunity have been identified, a key gene required to elicit rice-resistant protein Xa21 expression has not been identified [43]. As described earlier, control of BB involves the introduction of resistance genes to confer major gene-specific resistance against some pathogen races. However, this type of resistance frequently results in rapid changes in the pathogen diversity, and new races of the pathogen are able to overcome the deployed resistance [44,45].

In this study, we presented the comparison of genome-wide transcriptional patterns between two *Xoo* strains and used transcriptome profiling to identify genes that might be involved in the different pathotypes and that may be specific to the *Xoo* race. GO term enrichment analysis identified four remarkably enriched GO terms in the upregulated genes, and the expression of 44 genes (a total of 147 enriched genes) was considerably higher (log2 fold changes > 2) in the HB1009 (K3a race) strain than in the *Xoo* KACC10331 (K1 race) strain (Table S3). Among the 44 genes, 13 and seven genes were identified as transposase and hypothetical genes, respectively. In addition, six genes, identified as transcription regulators, showed a higher level of expression in the HB1009 (K3a race) strain than in the *Xoo* KACC10331 (K1 race) strain. Although the additional research is needed to determine the biological function of these genes, these results suggest that these enriched genes might be involved in host-specific immunity responses or facilitate virulence processes in the pathogen. *Xoo* virulence and the regulatory genes required for pathogenicity are commonly found in pathogenicity islands (PAIs) that encode for a type III secretion system (TTSS) assembled from *hrp* gene products [46,47,48]. In this study, several genes associated with the expression of *hrp* genes were significantly upregulated in HB1009. This result suggests that the expression levels of these *hrp* genes may differ for these two strains in rice varieties, since *hrp* genes were shown to be essential for bacterial pathogenicity in susceptible hosts and hypersensitive reaction (HR) induction in host and non-host plants in the plant pathogen [47,49]. TAL effectors also play key roles in host immune responses or virulence processes in the pathogen, and the genomic sequences revealed that Philippine (PXO99A), Japanese (MAFF311018), and Korean (KACC10331) strains contain 19, 17, and 15 TAL effector genes, respectively. In addition, Southern hybridization analysis revealed that the HB1009 strain contains 13 TAL effector genes in its chromosome (data not shown). Due to the repetitive structure of the TAL effector coding sequences, NGS-based transcriptome analysis is not sufficient to evaluate their expression. Although additional approaches such as site-directed mutagenesis of these TAL effectors and evaluation of host immunity responses are needed to facilitate the understanding of the function of these TAL effectors, transcriptome analysis revealed that the expression levels of these genes were slightly upregulated in the HB1009 strain; however, these genes did not show significantly different expression levels in the DEGseq analysis (*p*-value > 0.001, FALSE).

Zhang *et al.* [31] reported that the bacterial motilities were significantly different among the *Xoo* strains (C5, China race 5; P2 and P6, Philippines race 2 and 6, respectively), which showed different virulence patterns against various rice cultivars. This study also demonstrated that the genes encoding *Hrp* proteins and T3 effectors were significantly downregulated in C5, whereas no significantly different virulence pattern was observed among those strains in a virulence assay with various rice cultivars. Thus, they hypothesized that strong motility might compensate for the weaker expression of *Hrp* proteins in C5, which allows C5 to exhibit similar virulence levels with the other two *Xoo* strains. We also conducted a bacterial motility assay with the KACC10331 and HB1009 strains on semi-solid swarm plates. However, there was no significant difference in bacterial motility between the two strains (data not shown). These results suggest that the different virulence patterns of the two *Xoo* strains might not be due to differences in bacterial motility but, rather, might be due to differently expressed genes, including *hrp* and TCSs, as well as an enriched gene set according to the enrichment analysis between the two *Xoo* strains.

Although further studies are required to elucidate the relationship between the differently expressed genes and the *Xoo*–rice interaction, our findings should facilitate the identification of genes that are involved in this interaction, especially elicitors for the expression of the rice-resistance genes such as avrXa21, and will provide valuable insights to aid in developing future strategies to control BB caused by newly evolved strains such as HB1009 (K3a).

## 4. Materials and Methods

### 4.1. Bacterial Strains and Culture Conditions

The *Xoo* KACC10331 (K1 race) and HB1009 (K3a race) strains were obtained from the National Institute of Crop Science in Jeonju, Korea, and were cultured on YDC medium (2.0% d-glucose, 2.0% CaCO_3_, 1.0% yeast extract, and 1.5% agar) or in nutrient broth (3% beef extract, 5% peptone, and 1.5% agar; Difco) at 28 °C until 2.0 OD_600_. For total RNA isolation, the *Xoo* strains were washed twice and immediately transferred into XOM2 medium (0.18% xylose sugar, 670 mM d,l-methionine, 10 mM sodium l(+)-glutamate, 14.7 mM KH_2_PO_4_, 40 mM MnSO_4_, 240 mM Fe(III) EDTA, and 5 mM MgCl_2_, pH 6.5).

### 4.2. Virulence Assay

Seeds of the rice varieties were sown in a seedling nursery, and 30-day-old seedlings were transplanted. Leaves of 50-day-old (at the tillering stage) rice varieties were artificially infected with the *Xoo* strains (10^9^ cells·mL^−1^) by clipping the leaf tips with sterile scissors. The infected rice plants were then grown and maintained under greenhouse conditions (25–30 °C, 60% relative humidity). Distilled water was used as the control treatment. Twenty-one days after inoculation, the means values and standard deviations (SDs) of lesion lengths were calculated for each triplicate set of experiments.

### 4.3. Total RNA Isolation, Illumina Sequencing, and Data Analysis

Total RNA was extracted from a stationary phase culture (OD_600nm_ 0.8) using an RNeasy mini kit according to the manufacturer’s instructions (Qiagen, Hilden, Germany). Total RNA was further treated using RNase-free DNase set (Qiagen, Hilden, Germany) following manufacturer’s instructions to discard DNA contamination. Next-generation sequencing (NGS) library preparation and sequencing were performed with 1 μg of each total RNA using the Illumina HiSeq2000 platform in accordance with the manufacturer’s protocol (Illumina, Inc., San Diego, CA, USA). The raw data were deposited to the National Center for Biotechnology Information (NCBI) Sequence Reads Archive (SRA) with accession numbers SRP066131 (*Xoo* KACC10331) and SRP066133 (*Xoo* HB1009). Sequencing reads were processed by SolexaQA software [50] to control the quality of raw data. The trimmed reads were mapped to the *Xoo* KACC 10331 genome (NCBI accession no. NC_006834) using Bowtie aligner (http://bowtie-bio.sourceforge.net/index.shtml). The expression level was quantified by the reads per kilobase per million mapped reads (RPKM). DEGseq [25] was applied to identify differentially expressed genes (DEGs) with significance defined as a *p*-value less than 0.001. Gene Ontology (GO) term annotation and enrichment analysis of the upregulated and downregulated genes were carried out using Blast2GO software [26].

### 4.4. Quantitative Real-Time RT-PCR Assay

To validate the RNA-seq data, a subset of differentially expressed genes (DEGs) was verified by quantitative real-time RT-PCR (qRT-PCR). An independent set of cell cultures and total RNA samples from the two *Xoo* strains were prepared following the same protocol as for the Illumina analysis. The sequence of each gene was obtained from the *Xoo* 10331 database (http://www.ncbi.nlm.nih.gov) and used for designing primers by IDT (Integrated DNA Technologies, Coralville, IA, USA, https://eu.idtdna.com/Primerquest/Home/Index). RNA samples from three independent replicates were treated with DNase before cDNA synthesis. Quantitative real-time PCR analysis was performed using a RotorGene 6000 system (Qiagen, Hilden, Germany) in 25-μL reactions containing 12.5 μL SensiFAST SYBR No-ROX kit (Bioline, Sydney, Australia), 5 pmol of each primer (Table S5), and 25 ng of cDNA template. Reaction conditions were as follows: 3 min of holding at 95 °C followed by 40 cycles of 95 °C for 5 s, 60 °C for 10 s, and 72 °C for 15 s. The gene expression levels (arbitrary units) were normalized on the basis of transcript amounts of 16S RNA as an internal reference. The relative expression level of each gene is defined as Δ*C*t = *C*t_target_ − *C*t_16S_ to represent the difference between the transcript abundance of genes examined and the transcript abundance of 16S RNA.

## Figures and Tables

**Table 1:** Expression profiles of *hrp* genes in the *Xanthomonas* *oryzae* pv. *oryzae* KACC10331 and HB1009 strains. * True indicates significant differential expression (*p*-value < 0.001).

Gene	Locus Tag	Product	RPKM		DEGseq Analysis		
			10331	1009	log2 FC	*p*-Value	Signature *
*hpaF*	XOO0065	protein HpaF	13.28	26.08	0.97	0.06798	FALSE
*hrpF*	XOO0066	HrpF protein	58.03	98.96	0.77	0.00487	FALSE
*hpa4*	XOO0074	hypothetical protein	21.87	77.43	1.82	0.00000	TRUE
*hpaB*	XOO0075	protein HpaB	13.95	63.98	2.20	0.00000	TRUE
*hrpE*	XOO0076	hypothetical protein	24.12	55.05	1.19	0.00139	FALSE
*hrpD6*	XOO0077	protein HrpD6	23.53	55.18	1.23	0.00099	TRUE
*hrpD5*	XOO0078	protein HrpD5	14.07	46.30	1.72	0.00007	TRUE
*hpaA*	XOO0079	protein HpaA	8.59	37.25	2.12	0.00003	TRUE
*hrcS*	XOO0080	hypothetical protein	8.59	41.37	2.27	0.00000	TRUE
*hrcR*	XOO0081	type III secretion system protein	20.31	56.68	1.48	0.00009	TRUE
*hrcQ*	XOO0082	hypothetical protein	12.61	38.41	1.61	0.00058	TRUE
*hpaP*	XOO0083	hrpC3	6.95	29.32	2.08	0.00027	TRUE
*hrcV*	XOO0084	protein HrcV	6.78	23.74	1.81	0.00303	FALSE
*hrcU*	XOO0085	type III secretion system protein HrcU	5.77	17.98	1.64	0.01677	FALSE
*hrpB1*	XOO0086	protein HrpB1	10.00	30.18	1.59	0.00244	FALSE
*hrpB2*	XOO0087	protein HrpB2	8.59	51.32	2.58	0.00000	TRUE
*hrpB3* (*hrcJ*)	XOO0088	protein HrpB3	9.03	23.62	1.39	0.01673	FALSE
*hrpB4*	XOO0089	protein HrpB4	8.46	27.56	1.70	0.00228	FALSE
*hrpB5*	XOO0090	type III secretion system protein HrpB	6.72	23.82	1.83	0.00276	FALSE
*hrcN*	XOO0091	type III secretion system ATPase	5.06	19.22	1.93	0.00519	FALSE
*hrpB7*	XOO0092	protein HrpB7	5.91	22.69	1.94	0.00225	FALSE
*hrpB8* (*hrcT*)	XOO0093	protein HrpB8	6.60	26.50	2.00	0.00073	TRUE
*hrcC*	XOO0094	hypothetical protein	14.79	33.69	1.19	0.01258	FALSE
*hpa1*	XOO0095	protein Hpa1	15.64	41.50	1.41	0.00132	FALSE
*hpa2*	XOO0096	protein Hpa2	68.65	145.14	1.08	0.00000	TRUE
*hrpG*	XOO1379	HrpG protein	9.59	14.98	0.64	0.36548	FALSE
*hrpXct*	XOO1380	HrpX protein	8.26	8.83	0.10	0.98273	FALSE
*hpa3*	XOO4700	hypothetical protein	69.68	181.24	1.38	0.00000	TRUE

**Table 2:** Expression profiles of TCSs and *rax* gens in the *Xanthomonas* *oryzae* pv. *oryzae* KACC10331 and HB1009 strains. * True indicates significant differential expression (*p*-value <0.001).

Gene	Locus Tag	Product	RPKM		DEGseq Analysis		
			10331	1009	log2 FC	*p*-Value	Signature *
*ygiY*	XOO0057	two-component system sensor protein	23.33	43.05	0.77	0.03277	FALSE
–	XOO0336	two-component system sensor protein	6.14	25.69	1.96	0.00068	TRUE
*phoP*	XOO0423	two-component system regulatory protein	143.04	99.05	−0.64	0.00061	TRUE
*phoQ*	XOO0424	two-component system sensor protein	42.90	38.67	−0.26	0.41585	FALSE
–	XOO0519	two-component system sensor protein	8.56	37.48	2.02	0.00003	TRUE
–	XOO0520	two-component system regulatory protein	10.45	27.08	1.26	0.01107	FALSE
–	XOO0683	two-component response regulator	14.10	16.94	0.15	0.76601	FALSE
*tctD*	XOO1105	two-component system regulatory protein	58.18	117.85	0.91	0.00005	TRUE
*tctE*	XOO1106	two-component system sensor protein	21.90	52.13	1.14	0.00115	FALSE
*colS*	XOO1207	two-component system sensor protein	66.31	231.53	1.69	0.00000	TRUE
*colR*	XOO1208	two-component system regulatory protein	26.59	48.57	0.76	0.02578	FALSE
*creC*	XOO1477	two-component system sensor protein	69.24	97.83	0.39	0.08422	FALSE
*baeS*	XOO1558	two-component system sensor protein	3.69	13.48	1.76	0.02205	FALSE
*baeR*	XOO1559	two-component system regulatory protein	5.03	12.25	1.17	0.10729	FALSE
*pilR*	XOO1591	two-component system regulatory protein	21.08	18.95	−0.26	0.56344	FALSE
*pilS*	XOO1592	two-component system sensor protein	5.36	10.62	0.88	0.23802	FALSE
*regR*	XOO2227	two-component system regulatory protein	117.22	376.62	1.57	0.00000	TRUE
*regS*	XOO2228	two-component system sensor protein	43.39	165.10	1.82	0.00000	TRUE
–	XOO2322	two-component system regulatory protein	124.34	48.71	−1.46	0.00000	TRUE
–	XOO2323	two-component system sensor protein	16.32	17.80	0.02	0.97542	FALSE
*rrpX*	XOO2787	transcriptional regulator	13.09	21.30	0.59	0.23576	FALSE
–	XOO2797	two-component system sensor protein	10.54	12.89	0.18	0.76266	FALSE
–	XOO2798	two-component system regulatory protein	32.57	7.65	−2.20	0.00002	TRUE
*rpfC*	XOO2870	RpfC protein	18.06	27.96	0.52	0.22665	FALSE
*rpfG*	XOO2871	response regulator	39.38	52.65	0.31	0.30732	FALSE
–	XOO3527	two-component system regulatory protein	21.45	24.84	0.10	0.81159	FALSE
–	XOO3528	two-component system sensor protein	9.75	14.03	0.41	0.48663	FALSE
–	XOO3659	two-component system regulatory protein	114.21	209.67	0.77	0.00000	TRUE
*phoB*	XOO3666	two-component system regulatory protein	11.54	18.72	0.59	0.26937	FALSE
*phoR*	XOO3667	two-component system sensor protein	4.77	13.97	1.44	0.04384	FALSE
*torS*	XOO3709	two-component system sensor protein	7.90	34.31	2.01	0.00006	TRUE
–	XOO3710	two-component system regulatory protein	7.34	38.26	2.27	0.00001	TRUE
*colS*	XOO3762	two-component system sensor protein	8.93	13.62	0.50	0.41805	FALSE
*colR*	XOO3763	two-component system regulatory protein	59.32	42.52	−0.59	0.04006	FALSE
*kdpE*	XOO3842	two-component system regulatory protein	5.43	13.43	1.20	0.08655	FALSE
*kdpD*	XOO3843	two-component system sensor protein	10.16	13.90	0.34	0.56471	FALSE
–	XOO3870	two-component system regulatory protein	35.44	60.64	0.66	0.02655	FALSE
–	XOO3871	two-component system sensor protein	14.79	43.80	1.46	0.00032	TRUE
–	XOO3875	two-component system sensor protein	164.50	381.45	1.10	0.00000	TRUE
–	XOO3935	two-component system regulatory protein	11.04	15.52	0.38	0.49899	FALSE
–	XOO3936	two-component system sensor protein	16.87	19.88	0.13	0.79059	FALSE
*algR*	XOO4008	two-component system regulatory protein	26.39	59.48	1.06	0.00105	FALSE
*algZ*	XOO4009	two-component system sensor protein	13.28	19.44	0.44	0.38875	FALSE
–	XOO4201	two-component system sensor protein	20.73	26.41	0.24	0.57069	FALSE
*ntrC*	XOO4202	two-component system regulatory protein	8.72	15.56	0.72	0.22512	FALSE
*smeR*	XOO4341	two-component system regulatory protein	7.69	18.97	1.19	0.04211	FALSE
*ntrC*	XOO4483	two-component system regulatory protein	13.97	20.70	0.46	0.35612	FALSE
*ntrB*	XOO4484	two-component system sensor protein	40.25	58.94	0.44	0.13231	FALSE
*tcsR*	XOO4543	two-component system regulatory protein	7.25	18.38	1.23	0.04017	FALSE
raxP	XOO3397	sulfate adenylyltransferase subunit 2	10.46	18.71	0.73	0.18138	FALSE
*colS* (*raxH*)	XOO3534	two-component system sensor protein	7.85	14.60	0.78	0.20791	FALSE
*colS* (*raxR*)	XOO3535	two-component system regulatory protein	11.88	18.41	0.52	0.32541	FALSE
*raxB*	XOO3543	ABC transporter protein RaxB	8.04	15.86	0.87	0.15188	FALSE
*raxA*	XOO3544	membrane fusion protein RaxA	3.86	12.99	1.64	0.03244	FALSE
*raxST*	XOO3546	sulfotransferase RaxST	11.06	12.49	0.07	0.91282	FALSE

## Supplementary Materials

Supplementary materials can be found at http://www.mdpi.com/1422-0067/17/2/259/s1.

## Abbreviations

*Xoo*: *Xanthomonas**oryzae* pv. *oryzae*
BB: bacterial blight
*hrp*: hypersensitive response and pathogenicity
TCSs: two-component regulatory systems
PRRs: pattern recognition receptors
QS: quorum sensing
DSF: diffusible signal factor
*rax*: required for activation of XA21-mediated immunity
TTSS: type III secretion system
DEGs: differentially expressed genes
qRT-PCR: quantitative real-time RT-PCR
